# The epidermal growth factor receptor variant type III mutation frequently found in gliomas induces astrogenesis in human cerebral organoids

**DOI:** 10.1111/cpr.12965

**Published:** 2020-12-06

**Authors:** Hyun‐Mun Kim, Sang‐Hyeok Lee, Jaejoon Lim, Jongman Yoo, Dong‐Youn Hwang

**Affiliations:** ^1^ Department of Biomedical Science Graduate School of CHA University Seongnam Korea; ^2^ Department of Neurosurgery Bundang Medical Center CHA University Seongnam Korea; ^3^ Department of Microbiology School of Medicine CHA University Seongnam Korea

**Keywords:** astrogenesis, cancer modelling, cerebral organoid, EGFRvIII, glioblastoma, temozolomide

## Abstract

**Objectives:**

The epidermal growth factor receptor variant type III (*EGFRvIII*) is the most common mutation of EGFR in glioblastoma multiforme (GBM) and is found in approximately 25% of all GBMs. Intriguingly, *EGFRvIII* is mostly found in GFAP^+^ astrocytic tumour cells in the brain, suggesting connection of *EGFRvIII* to astrogenesis. In this study, we explored whether *EGFRvIII* mutation facilitates astrogenesis in human development setting.

**Materials and methods:**

Using CRISPR‐Cas9, we generated *EGFRvIII* mutations in H9‐hESCs. Wild type (*wt*) H9‐hESCs were used as an isogenic control. Next, we generated cerebral organoids using the *wt* and *EGFRvIII‐*hESCs and examined the astrogenic differentiation of the brain organoids.

**Results:**

*EGFRvIII‐*organoids showed abundant astrocytes (GFAP^+^, S100β^+^), while no astrocytes were detected in *wt* hESC‐derived organoids at day 49. On the contrary, TUJ1^+^ neurons were more abundant in the *wt*‐organoids than the *EGFRvIII*‐organoids. This result suggested that constitutively active *EGFRvIII* promoted astrogenesis at the expense of neurogenesis. In addition, the *EGFRvIII*‐organoids were larger in size and retained more Ki67^+^ cells than *wt‐*organoids, indicating enhanced cell proliferation by the mutation. The *EGFRvIII‐*organoids displayed massive apoptotic cell death after treatment with temozolomide and hence, could be used for evaluation of anti‐GBM drugs.

**Conclusions:**

*EGFRvIII* mutation‐induced astrogenesis and massive cell proliferation in a human brain development model. These results provide us new insights into the mechanisms relating *EGFRvIII* mutation‐mediated gliogenesis and gliomagenesis.

## Graphical abstract

The epidermal growth factor receptor variant type III (EGFRvIII) mutation mostly found in high‐grade astrocytoma in the brain, suggesting connection of EGFRvIII to astrogenesis. Using human brain organoids, we demonstrated that EGFRvIII mutation facilitated astrogenesis at the expense of neurogenesis and cell proliferation. In summary, this study shows for the first time the close connection between EGFRvIII mutation and gliogenesis in human system.

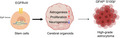

## 1 INTRODUCTION

A growing body of evidence suggests that tumorigenesis is closely linked to developmental processes. Therefore, study of the function of tumour drivers (ie, gene mutations) in the setting of developmental biology may help us to better understand the process of tumorigenesis.^1^, ^2^ Brain tumours are generally classified into glioma and non‐glioma. There are three principle types of glioma: astrocytoma, oligodendroglioma and glioblastoma (GBM).^3^ Among them, GBM is the most frequent and aggressive primary brain tumour, accounting for more than 60% of all brain tumours.^4^, ^5^, ^6^ Unfortunately, despite intensive research efforts over the past few decades, the median survival rate has not changed significantly.^7^ This may be at least partly attributed to the lack of an appropriate cancer model to study the generation of GBM.

In vitro 2‐dimentional (2‐D) culture models have frequently been used in studies of developmental biology and tumorigenesis due to their convenience. However, these 2‐D models do not accurately mimic the diversity of cell types and cell‐to‐cell and cell‐to‐extracellular environment interactions, often failing to recapitulate conditions seen in vivo.

To bridge this translational gap, patient‐derived xenograft (PDX) models have recently been developed. In PDX models, samples of tumour tissues from human cancer patients are grafted onto immunocompromised animals and used to study new treatments or basic biology. However, in addition to significant cost‐ and labour‐intensiveness, results from PDX models often fail to predict outcomes in human clinical trials.^8^, ^9^, ^10^

Brain organoids have recently emerged as a promising 3‐D model that recapitulates many aspects of the human brain.^11^, ^12^ In this study, we used brain organoid models to investigate the role of a tumour driver mutation in GBM and to evaluate the therapeutic effects of temozolomide, an anti‐cancer drug. Using CRISPR/Cas9,^13^ we introduced an epidermal growth factor receptor variant type III (*EGFRvIII*) mutation into hESCs, a mutation that is found in approximately 25% of all GBM patients.^14^, ^15^ We found that the *EGFRvIII* mutation, which generates a ligand‐independent and constitutively active form of EGFR,^16^ promoted astrogenesis and also induced massive cell proliferation which was blocked by temozolomide, a drug used for the treatment of GBM.

This study adds new insights to the process of gliomagenesis and provides a useful platform to evaluate the efficacy of other anti‐GBM drugs.

## 2 MATERIALS AND METHODS

### 2.1 ESC culture

Human H9 (WA09) embryonic stem cells purchased from WiCell Research Institute, Inc hESCs were cultured on Matrigel (Thermo Fisher Scientific)‐coated culture dishes in the TeSR^™^‐E8^™^ medium (STEMCELL Technologies). Cells were passaged every 5 days by treating with 0.5 mmol/L EDTA (Thermo Fisher Scientific) for 3 minutes at 37°C and transfering at 1:20 ratio onto fresh matrigel‐coated plates containing TeSR^™^‐E8^™^ medium with 10 μmol/L Y‐27632 (Tocris). Next day, we changed the medium with fresh TeSR^™^‐E8^™^ medium without Y‐27632.

### 2.2 CRISPR/Cas9‐mediated genome editing of hESC

CRISPR/Cas9‐mediated genome editing of hESCs was performed as follows: hESCs were dissociated into single cells using Accutase Dissociation Reagent (Thermo Fisher Scientific) and subjected to electroporation with 30 μg of Cas9 protein and 20 μg of sgRNA. Cells were then cultured on matrigel‐coated culture dishes and maintained in TeSR^™^‐E8^™^ medium. Y27632 (10 μmol/L) was added to the medium for the first 24 hours. After 5 days, the colonies were dissociated into single cells and seeded onto fresh matrigel‐coated dishes at a low density to obtain clones. Individual colonies grown for 5 days were transferred into each well of 48 well plates and expanded in TeSR^™^‐E8^™^ medium. Table S1 contains the list of sgRNA used in this study.

### 2.3 RNA extraction, reverse transcription and RT‐PCR

Total RNAs were extracted using the Nucleospin RNA kit (MACHEREY‐NAGEL), according to the manufacturer's protocol. RNA concentration was measured using the NanoDrop Spectrophotometer (Thermo Fisher Scientific) and cDNA was synthesized using a ReverTra Ace^®^ qPCR RT kit (TOYOBO). The resulting cDNA was amplified by the polymerase chain reaction (PCR) using EGFR primers (Table S1).

### 2.4 Differentiation into the three germ layer

hESCs were detached with 2 mg/mL collagenase Type IV (Worthington Biochemical) for 30 minutes at 37°C. The detached cells were centrifuged at 300 g for 5 minutes and plated on Petri dishes containing DMEM/F12, 20% Knockout Serum Replacement, 1% NEAA and 55 μmol/L 2‐Mercaptoethanol (all from the Thermo Fisher Scientific) for embryoid body (EB) formation. After 7‐day culture, the EBs were attached to martigel‐coated dishes and subjected to spontaneous differentiation in DMEM/F12 medium containing 10% FBS, and 1% Penicillin/Streptomycin (all from the Thermo Fisher Scientific) for 1‐2 weeks.

### 2.5 Immunocytochemistry

Undifferentiated cells and differentiated cells into the three germ layer fixed in 4% paraformaldehyde (PFA) for 30 minutes at room temperature (RT), washed 2‐3 times with 1X phosphate‐buffered saline (PBS). Fixed cells permeabilized with 0.2% Triton‐X100/1X PBS for 10 minutes, were blocked with 5% normal goat serum for 1 hours at RT, followed by subsequent incubation with primary and secondary antibodies for 3 hours and 1 hour, respectively, at RT. The following primary antibodies were used: OCT4 (Santa Cruz; sc‐5279, 1:500), SSEA4 (Santa Cruz; sc‐21704, 1:500), TRA1‐81(Millipore; MAB4381, 1:500), SMA (Agilent; M0851, 1:500) and AFP (Agilent; A0008, 1:500). Secondary antibodies conjugated with Alexa‐488 and ‐594 (Thermo Fisher Scientific; 1:500), were used depending on the primary antibody used. DAPI (4',6‐Diamidino‐2‐Phenylindole, Dihydrochloride) was used for counter‐staining at a concentration of 1.0 μg/mL (Table S2).

### 2.6 Generation of brain organoids

A total of 10 000 cells were plated into each well of an ultra‐low‐attachment 96‐well plate (Thermo Fisher Scientific) containing E6 medium with 4 ng/mL bFGF (CHA Meditech) and 50 μmol/L Y27632. EBs were formed and maintained in the 96‐well plate for 6 days, followed by transferring into a Petri dish containing neural induction medium. Neural induction medium consisted of DMEM/F12, 1% N2 supplement, 1% NEAA, 1% GlutaMAX (all from Thermo Fisher Scientific) and heparin (1 μg/mL; Sigma‐Aldrich). On day 11, EBs were embedded in droplets of matrigel and then solidified at 37°C. Embedded EBs were subsequently incubated in neural differentiation medium containing 1:1 mixture of DMEM/F12 and Neurobasal, 1% N2 supplement, 2% B27 supplement Minus Vitamin A, 0.5% NEAA, 1% GlutaMAX, 25 μmol/L 2‐Mercaptoethanol (all from Thermo Fisher Scientific) and human Insulin (2.5 μg/mL; Sigma‐Aldrich). The tissue droplets in 6‐cm Petri dishes were cultured in stationary phase for 4 days, and then transferred to an orbital shaker (Daihan Scientific) that rotated continuously at 80 rpm. During the rotation culture, the neural differentiation medium was changed to a fresh neural differentiation medium that contained 2% B27 with ascorbic acid (Thermo Fisher Scientific).

### 2.7 Histology and imaging

Organoids were fixed in 4% paraformaldehyde (PFA) for 30 minutes at RT, washed 2‐3 times with 1× PBS and subject to dehydration overnight at 4°C with 30% sucrose solution. The dehydrated organoids were frozen in Optimal cutting temperature (OCT) compound (Sakura^®^ Finetek), followed by sectioning (10‐15 μm thick) using Leica CM3050 S Research Cryostat (Leica). Sections on the slides were permeabilized with 0.2% Triton‐X100/1XPBS for 10 minutes, were blocked with 5% normal goat serum for 30 minutes at RT, followed by subsequent incubation with primary and secondary antibodies for 3 hours and 1 hour, respectively, at RT. The following primary antibodies were used: SOX2 (Millipore; MAB4343, 1:200), PAX6 (BioLegend; 901301, 1:200), NeuN (Millipore; MAB377, 1:200), MAP2 (Millipore; AB5622, 1:200), Nestin (Millipore; MAB5326, 1:200), TUJ1 (Convance; MMS‐435P, 1:500), GFAP (Thermo Fisher Scientific; MA5‐12023, 1:200; Abcam, ab68428, 1:200), S100β (Novus Biologicals; NBP1‐87102, 1:200), Ki67 (BD Biosciences; 550609, 1:500) and cleaved caspase‐3 (Cell Signaling; 9661, 1:500). Secondary antibodies conjugated with Alexa‐488 and ‐594 (Thermo Fisher Scientific; 1:500) and DAPI were stained. Fluorescent images were captured using a Zeiss LSM‐700 confocal microscope (Zeiss; Table S2).

### 2.8 Temozolomide treatment

Brain organoids were grown for 50 days and then treated with 1 mmol/L Temozolomide for 10 days. DMSO was used as a negative control. Medium containing Temozolomide was changed daily during treatment period. The treated brain organoids were frozen and subjected to immunostaining with antibodies against cleaved caspase 3 (Cell Signaling Technology) and Ki67 (BD Biosciences).

### 2.9 Statistical analysis

Statistical analysis was performed using the graphpad prism Version 5 program. Unpaired two‐tailed Student's t test was used to compare two groups, while one‐way ANOVA with Turkey's test was used to compare the multiple groups. Only *P*‐values smaller than .05 were considered statistically significant.

## 3 RESULTS

### 3.1 Generation of brain organoids

We generated cerebral organoids from H9‐hESCs using a protocol established by the Knoblich group^11^ with one modification: for the first 6 days of culture, we used E6 medium containing 4 ng/mL bFGF and 50 μmol/L Y27632 instead of DMEM/F12, 20% knockout serum replacement (KSR), 3% FBS, 1% Glutamax, 1% NEAA, 0.0007% 2‐Mercaptoethanol, 4 ng/mL bFGF and 50 μmol/L Y27632. This modification resulted in the generation of EB more efficiently.

**FIGURE 1:**
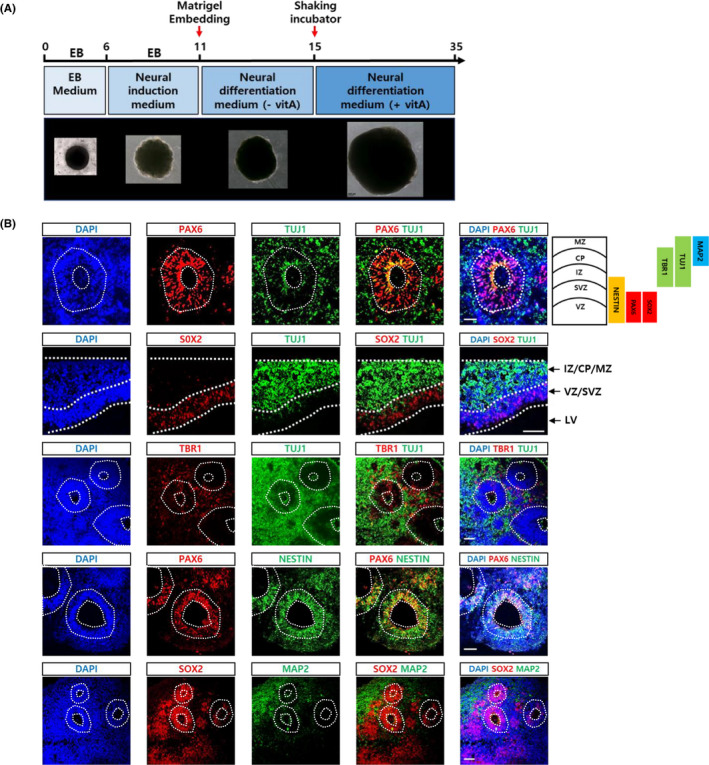
Generation of cerebral organoids. A, Schedule of brain organoid generation using an unguided method.^10^ The shape and relative sizes of brain organoids at each step were shown at the bottom. B, Brain organoids at 6 weeks of culture were sectioned and analysed for the expression of various neural markers: Neural progenitor markers (PAX6, Nestin and SOX2), early neuronal markers (TUJ1), mature neuronal marker (MAP2) and postmitotic cortical neuronal marker (TBR1) were shown. Scale bar, 50 μm. Layers and representative markers of human cerebral cortex were shown on the *top & right side*. CP, cortical plate; IZ, intermediate zone; LV, lateral ventricles; MZ, marginal zone; SVZ, subventricular zone; VZ, ventricular zone

Matrigel embedding was performed at day 11 of organoid culture, and shaking was initiated on day 15. As expected, the size of brain organoids increased as the incubation period went on. The detailed structures of the cerebral organoids were analysed at day 35‐40 (Figure 1A).

Several structures containing epithelial‐type cells surrounding the ventricle‐like space were frequently detected. In particular, cortical structures composed of ventricular and subventricular zones (VZ and SVZ) marked by SOX2 and PAX6 antibodies were often evident. NESTIN expression mostly overlapped with SOX2 and PAX6, but its expression extended further into the intermediate zone (IZ). TUJ1 (an early neuronal marker) marked the IZ, cortical plate (CP) and marginal zone (MZ). TBR1 (a deep‐layer cortical marker) was expressed in the IZ and CP. Finally, MAP2 (a mature marker) was mostly detected in the CP and MZ (Figure 1B).

These results suggested that our cerebral organoids recapitulated the typical features of early cerebral development.

### 3.2 Generation of the *EGFRvIII* mutation

**FIGURE 2:**
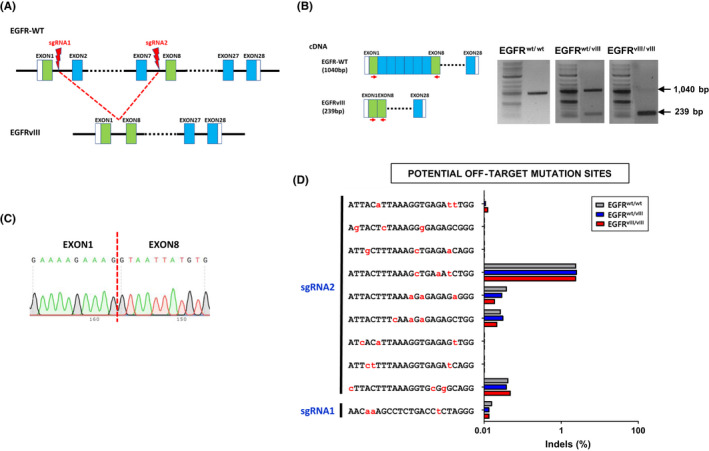
Generation of *EGFRvIII*‐hESC lines by CRISPR/Cas9 genome editing. A, Scheme of generation of *EGFRvIII* mutation by CRISRP/Cas9 system. Two sgRNA binding sites, one in intron 1 and the other in intron 7 of wild type EGFR locus, were indicated. B, Confirmation of monoallelic and biallelic *EGFRvIII* mutations was confirmed by RT‐PCR using primer pairs recognizing exon1 and exon 8: Primers were indicated as red arrows. Wild type *EGFR* cDNA produces 1040 bp PCR product, while *EGFRvIII* cDNA produces 239 bp DNA band. C, Sequencing analysis confirmed the deletion of exon 1 through exon 7 in *EGFRvIII* mutation. D, Targeted deep‐sequencing analysis revealed undetectable off‐target mutations in both *EGFR*‐edited clones, *EGFR^wt/vIII^* and *EGFR^vIII/vIII^*. Prediction of potential off‐target sites with up to three mismatches with the two sgRNA target sequences (sgRNA1‐targeting intron1 and sgRNA2‐targeting intron7 sequences) was performed with CRISPR RGEN Tools website (http://www.rgenome.net/about/): Our *EGFR* sgRNA target sequences had no potential off‐target sites with one or two mismatches, but had ten potential off‐target sites with three mismatches (one site in intron 1 and 9 sites in intron 7). The mismatched nucleotides were denoted in red and small capital letters in each sequence

*EGFRvIII* is the most common mutation of EGFR and is found in ~25% of all cases of GBM. The mutation is characterized by the loss of exons 2‐7 of the *EGFR* gene, resulting in an in‐frame deletion of 267 amino acids in the extracellular domain. *EGFRvIII* has not been detected in normal brains but is found in glioma. To explore whether the *EGFRvIII* mutation alone could promote gliogenesis and cell proliferation, we first sought to generate the mutation by deleting exon 2 to 7 of *EGFR* in H9‐hESCs using CRISPR/Cas9 (Figure 2A). As a result of genome editing, we generated *EGFR^wt/vIII^*‐ and *EGFR^vIII/vIII^*‐hESC lines which had a monoallelic and biallelic *EGFRvIII* gene, respectively. These mutations were confirmed via RT‐PCR and DNA sequencing (Figure 2B,C).

To assess for potential off‐target editing effects, we compared the sgRNAs targeting intron 1 and 7 of the *EGFR* gene, sgRNA1 and 2, respectively, to the whole human genome. We found no off‐target sequences that matched these sgRNAs completely or with a 1‐nucleotide mismatch. However, a total of ten off‐target sequences had a 3‐base mismatch with one of the two sgRNAs, as shown in Figure 2D. Deep sequencing of the *EGFRvIII* (*EGFR^wt/vIII^* and *EGFR^vIII/vIII^*)‐hESC lines showed that no off‐target mutations were generated at any of these 10 3‐nt mismatching sequences after genome editing (Figure 2D).

**FIGURE 3:**
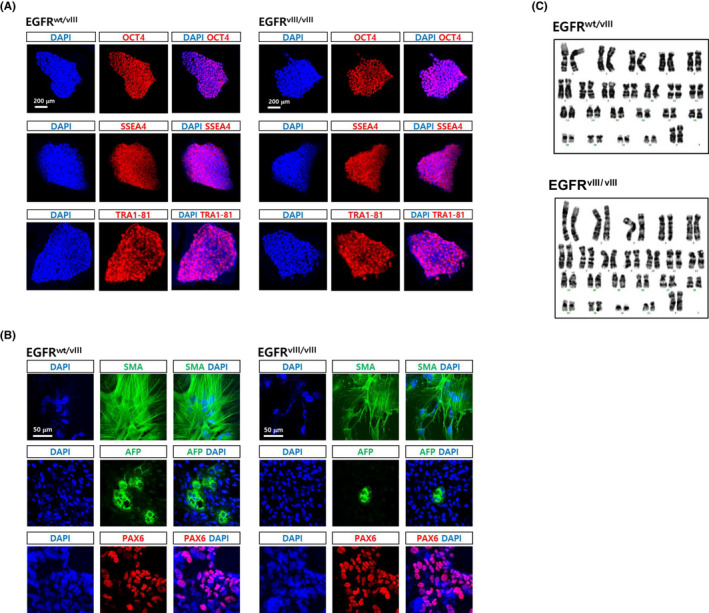
Characterization of *EGFRvIII‐*hESC clones. A, Both monoallelic and biallelic *EGFRvIII* clones were immunostained with antibodies recognizing typical hESC markers, Oct4, SSEA4, Oct4 and Tra1‐81. DAPI staining (blue) were also performed to expose the presence of cells. Scale bar, 200 μm. B, The *EGFRvIII* clones were spontaneously differentiated into derivatives of the three germ layers for 3 weeks via EB formation. Smooth muscle actin (SMA), alpha‐fetoprotein (AFP) and PAX6 were detected by immunostaining as markers for mesoderm, endoderm and ectoderm, respectively. Scale bar, 50 μm. C, G‐banding analysis of the *EGFRvIII* clones was performed to examine the presence of gross chromosomal abnormality

We confirmed that the *EGFRvIII*‐hESC lines retained pluripotency through verifying expression of typical pluripotency markers (OCT4, SSEA4 and TRA1‐81; Figure 3A). Further, we found that the edited cell lines could still be differentiated into cells of all three lineages, mesoderm (smooth muscle actin, SMA), endoderm (alpha‐fetoprotein, AFP) and ectoderm (PAX6; Figure 3B). G‐banding analysis revealed no gross chromosomal aberrations in the *EGFRvIII*‐hESC clones (Figure 3C).

Taken together, these studies suggested that we successfully generated monoallelic (*EGFR^wt/vIII^*) and biallelic (*EGFR^vIII/vIII^*) *EGFRvIII‐*hESC lines that retained pluripotency, which could be used as a model of the role of the *EGFRvIII* mutation in gliogenesis.

### 3.3 *EGFRvIII*‐cerebral organoids

**FIGURE 4:**
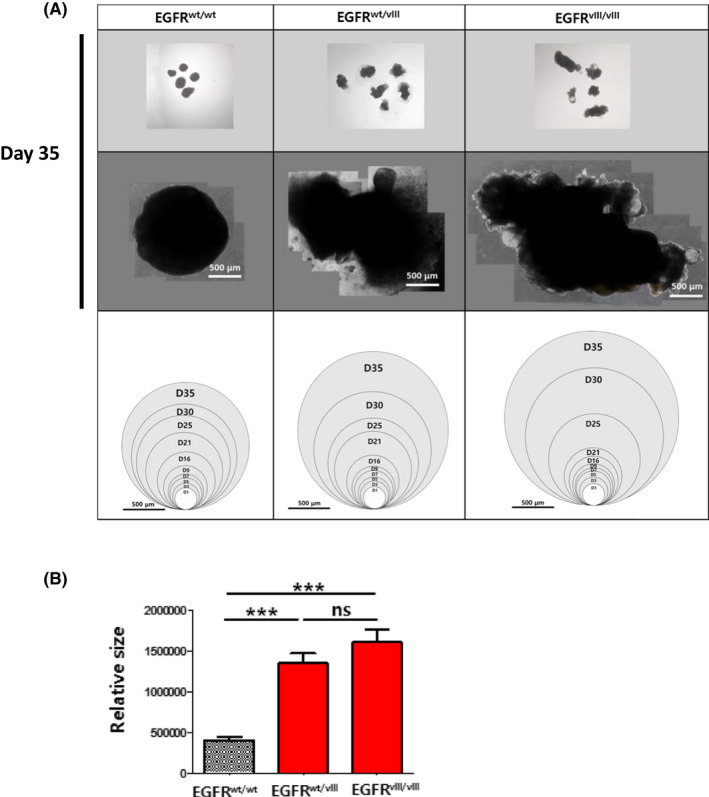
Size comparison of normal and *EGFRvIII‐*brain organoids. A, The bright‐field images of whole‐brain organoids were taken from day 1 through day 35. The representative images of brain organoids at day 35 were shown on *top and middle panels*. Total areas of brain organoids at individual days were measured by imagej software and were denoted as a circle corresponding to the area (*bottom panels*). Scale bar, 500 μm. B, The sizes of brain organoids of each group were shown in a graph. Results are presented as mean ± SEM. *P < .05; **P < .01; ***P < .001; ns, not significant

We generated cerebral organoids using monoallelic and biallelic *EGFRvIII*‐hESCs. Wild type (*wt*) H9‐hESCs (*EGFR^wt/wt^*‐hESCs) were used as an isogenic control. The size of both brain organoids that were generated from the *EGFRvIII*‐hESCs (*EGFR^wt/vIII^* and *EGFR^vIII/vIII^*‐hESCs) was much larger than those generated from *wt*‐hESCs (Figure 4A,B).

**FIGURE 5:**
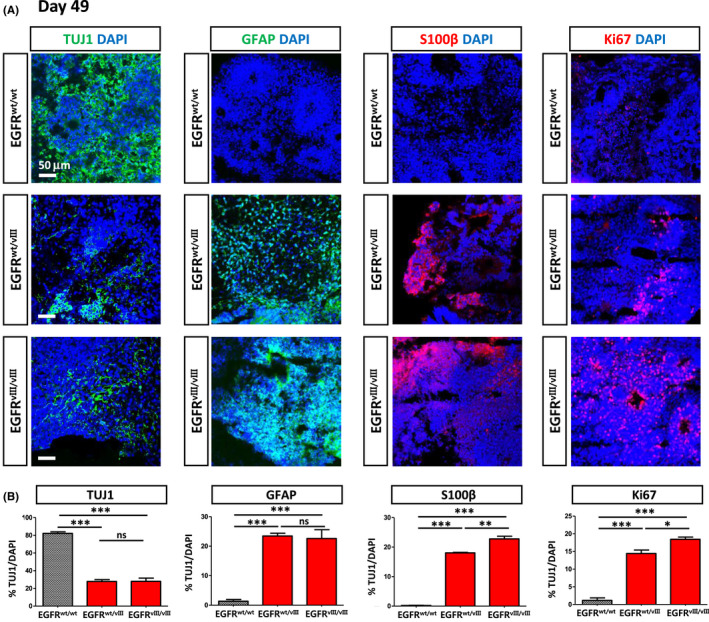
Immunofluorescence images of brain organoids. A, Brain organoids were generated using normal and *EGFRvIII*‐hESCs and were compared for the expression of representative markers for neurons (TUJ1), astrocytes (GFAP and S100β) and proliferating cells (Ki67) at day 49 by immunostaining. Scale bar, 50 μm. B, Expression of each marker among different organoids was quantified as % marker/DAPI. For this, the numbers of TUJ1^+^, GFAP^+^, S100β^+^ and Ki67^+^ cells in each brain organoid were quantified among total cells (DAPI^+^). Three independent experiments were performed

We used immunostaining to analyse the expression of representative neuronal (TUJ1) and glial (GFAP and S100β) markers. Beta‐tubulin III (TUJ1) was extensively detected in *wt*‐organoids, but significantly fewer TUJ1^+^ cells were detected in both *EGFR^wt/vIII^* and *EGFR^vIII/vIII^*‐organoids (Figure 5A,B, left‐most column). Two astrocyte markers, GFAP and S100β, were not detected in *wt*‐organoids. In contrast, the expression of GFAP and S100β was prominent in organoids derived from *EGFR^wt/vIII^* and *EGFR^vIII/vIII^*‐hESCs (Figure 5A,B, the second and third columns). GFAP immunostaining of the whole‐brain organoids showed extensive signal in *EGFR^wt/vIII^* and *EGFR^vIII/vIII^*‐organoids compared to sparse signal from *wt*‐organoids (Figures S1 and S2).

The number of proliferating cells (Ki67^+^ cells) was significantly higher in both *EGFR^wt/vIII^* and *EGFR^vIII/vIII^*‐organoids relative to *wt*‐organoids (Figure 5A,B, the right‐most column), consistent with the larger size of *EGFR^wt/vIII^* and *EGFR^vIII/vIII^*‐organoids compared with *wt*‐organoids.

Together, these results suggest that the *EGFRvIII* mutation promoted astrogenesis and massive cell proliferation in this organoid model.

### 3.4 Temozolomide treatment of the *EGFRvIII*‐brain organoids

**FIGURE 6:**
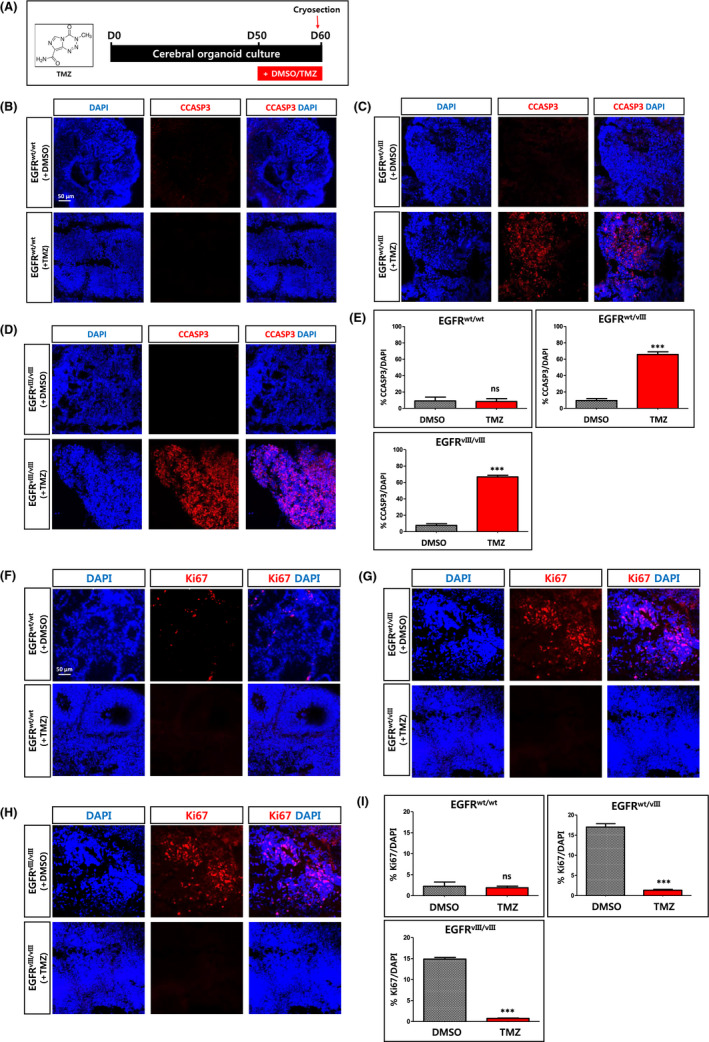
Effects of Temozolomide (TMZ) on *EGFRvIII*‐brain organoids. A, Scheme of treatment of brain organoids with Temozolomide (TMZ). As a negative control, DMSO was used. B‐D, After treatment with TMZ or DMSO for 10 days, the brain organoids were analysed by immunostaining using antibody recognizing cleaved caspase 3 (CCASP3). Brain organoids were generated using *wt* H9‐hESCs (B), monoallelic *EGFRvIII* (*EGFR^wt/vIII^*)‐hESCs (C) and biallelic *EGFRvIII* (*EGFR^vIII/vIII^*)‐hESCs (D). Scale bar, 50 μm. E, The percentages of CCASP3‐positive cells among total cells (DAPI^+^) were shown as graph. Results are presented as mean ± SEM. ***P < .001. N = 3 independent experiments. F‐H, After treatment with TMZ or DMSO for 10 days, the extent of proliferating cells in the brain organoids was analysed by immunostaining using antibody recognizing Ki67. Brain organoids were generated using *wt* H9‐hESCs (F), monoallelic *EGFRvIII* (*EGFR^wt/vIII^*)‐hESCs (G) and biallelic *EGFRvIII* (*EGFR^vIII/vIII^*)‐hESCs (H). Scale bar, 50 μm. I, The percentages of Ki67‐positive cells among total cells (DAPI^+^) were shown as a graph. Results are presented as mean ± SEM. **P < .01; ***P < .001; ns, not significant. N = 3 independent experiments

Temozolomide (TMZ), an alkylating agent, is a chemotherapy drug that has been used to treat GBM and anaplastic astrocytoma. To test whether brain organoids are a good model to evaluate the efficacy of anti‐cancer drugs, we treated the *EGFRvIII*‐brain organoids with 1 mmol/L TMZ for 10 days (between days 50 and 60, Figure 6A).

Immunostaining of the brain sections showed that cleaved caspase 3 (CCASP3), an apoptotic marker, was extensively detected in *EGFRvIII* (both *EGFR^wt/vIII^* and *EGFR^vIII/vIII^*)‐organoids, but not in *wt*‐organoids after TMZ treatment (Figure 6B‐E).

As seen in Figure 5 (at day 49), massive proliferation of Ki67^+^ cells was detected in *EGFRvIII* (both *EGFR^wt/vIII^* and *EGFR^vIII/vIII^*)‐organoids, but not in *wt*‐organoids at day 60 without TMZ treatment. On the other hand, a 10‐day treatment with TMZ robustly reduced the number of Ki67^+^ cells in EGFR^wt/vIII^ and EGFR^vIII/vIII^‐organoids (Figure 6F‐I). This observation was in line with the significant increase in cell death in *EGFRvIII*‐organoids, but not in *wt*‐organoids, after TMZ treatment, as seen in Figure 6B‐E.

Taken together, these results show that the great increases in cell proliferation due to the *EGFRvIII* mutation were reversed by treatment with TMZ.

## 4 DISCUSSION

The outcome of any developmental process is the production of mature cells of various lineages that contribute to the physiological function of tissues. In malignancy, the processes that generate the fully matured cells are subverted, resulting in the aberrant production of great numbers of lineage‐associated cells that eventually become malignant. Emerging from these observations is a molecular and functional interrelationship of the genetic drivers (ie, mutations) at the interface of development and malignancy.

One of the most common genetic aberrations in GBM is an *EGFR* mutation called *EGFRvIII*, caused by in‐frame deletion of exons 2‐7 (amino acids 6‐273) of the *EGFR* gene. Due to the large deletion of exons 2‐7, a significant portion of the extracellular domain of EGFR is lost, resulting in a constitutively active tyrosine kinase. Intriguingly, the *EGFRvIII* mutation is prevalent in gliomas but not in other types of brain tumours, suggesting a specific role of *EGFRvIII* in gliomagenesis.

In this study, using a brain organoid model, we explored whether the *EGFRvIII* mutation‐induced gliogenesis and contributed to gliomagenesis. To this end, we used CRISPR/Cas9‐genome editing to generate monoallelic (*EGFR^wt/vIII^*) and biallelic (*EGFR^vIII/vIII^*) conditions by removing exons 2‐7 of the *EGFR* gene from one or both alleles of hESCs, respectively, and generated brain organoids using the resulting cell lines. Wild type hESCs were used as an isogenic control.

We observed that *EGFRvIII* (both *EGFR^wt/vIII^* and *EGFR^vIII/vIII^*)‐organoids grew larger than *wt‐*organoids after 25 days of culture. In addition to the increased size, the shape of the *EGFRvIII*‐organoids became irregular, but *wt‐*organoids retained a round shape. Consistently, we detected a substantial number of proliferating (Ki67^+^) cells in *EGFRvIII*‐organoids, but not in *wt‐*organoids at day 49 of culture. This result suggested that the *EGFRvIII* mutation promoted abnormal proliferation of the cells in the *EGFRvIII*‐organoids, which may eventually lead to malignancy.

Strikingly, cells expressing astrocyte markers (GFAP^+^ or S100β^+^ cells), were abundant throughout the *EGFR^wt/vIII^* and *EGFR^vIII/vIII^*‐organoids, but no such cells were detected in *wt*‐organoids at day 49. In contrast, TUJ1^+^ neurons were much more abundant in the brain organoids derived from *wt* (*EGFR^wt/wt^*)‐hESCs relative to the *EGFRvIII‐*hESCs. This result suggested that the constitutive activation due to *EGFRvIII* promoted astrogenesis at the expense of neurogenesis. In line with our result, previous reports have demonstrated that stimulation of EGFR signalling induces preferential differentiation toward glial lineages both in vitro and in vivo experiments in rats.^17^, ^18^, ^19^

Our human brain organoid model showed that *EGFRvIII* mutation tended to steer neural differentiation toward gliogenesis (ie, astrogenesis) and induced massive proliferation, which may contribute to the formation of glioma. To our knowledge, this is the first study directly showing a connection between the *EGFRvIII* mutation and gliogenesis/gliomagenesis in a human system, let alone in brain organoids.

More than 90% of GBMs are primary brain tumours that develop rapidly de novo in adults. Recent studies have demonstrated that GBMs arise from neural stem cells (NSCs) that acquire driver mutations in the subventricular zone (SVZ), both in rodents and humans.^20^, ^21^ Therefore, it is plausible that the *EGFRvIII* mutation occurring in NSCs in the SVZ induces astrogenesis at the expense of neurogenesis and leads to active proliferation, eventually causing GBM. Likewise, cancer stem cells harbouring the *EGFRvIII* mutation may generate GBM.

Because of their intense proliferation, we examined whether *EGFRvIII*‐organoids could be used as a model to test the efficacy of GBM drugs. We found that treatment with TMZ significantly induced apoptosis in *EGFRvIII‐*organoids, but not in *wt‐*organoids. Therefore, the *EGFRvIII*‐brain organoids may provide a useful system to evaluate the efficacy of potential anti‐glioma drugs, especially those that target the *EGFRvIII* receptor.

This work suggests a clear role for *EGFRvIII* in gliomagenesis, laying a foundation for further investigation of its role in various aspects of glioma such as initiation and invasiveness, as well as in the development of novel anti‐glioma therapeutics.

## CONFLICT OF INTEREST

All authors of this paper declare no potential conflicts of interest.

## AUTHOR CONTRIBUTIONS

H.K., J.L., J.Y., D.H. designed the project. H.K. performed development of methodology. H.K., D.H. performed acquisition of data, analysis and interpretation and wrote the paper. H.K., S.L., J.L., J.Y., D.H. performed review, and/or revision of the manuscript. S.L. supported technical, or material support. D.H. performed study supervision.

## Supporting information

Fig S1‐S2

Table S1

Table S2

## Data Availability

The data that support the findings of this study are available from the corresponding author upon reasonable request.
