# Supplementary material for: The epidermal growth factor receptor variant type III mutation frequently found in gliomas induces astrogenesis in human cerebral organoids
Source: Cell Prolif. 2020 Dec 6;54(2):e12965. doi: 10.1111/cpr.12965 (PMC7848959; doi:10.1111/cpr.12965)
Supplement: Supplementary file 1 — Fig S1‐S2 [file CPR-54-e12965-s001.pptx]

## Slide 1
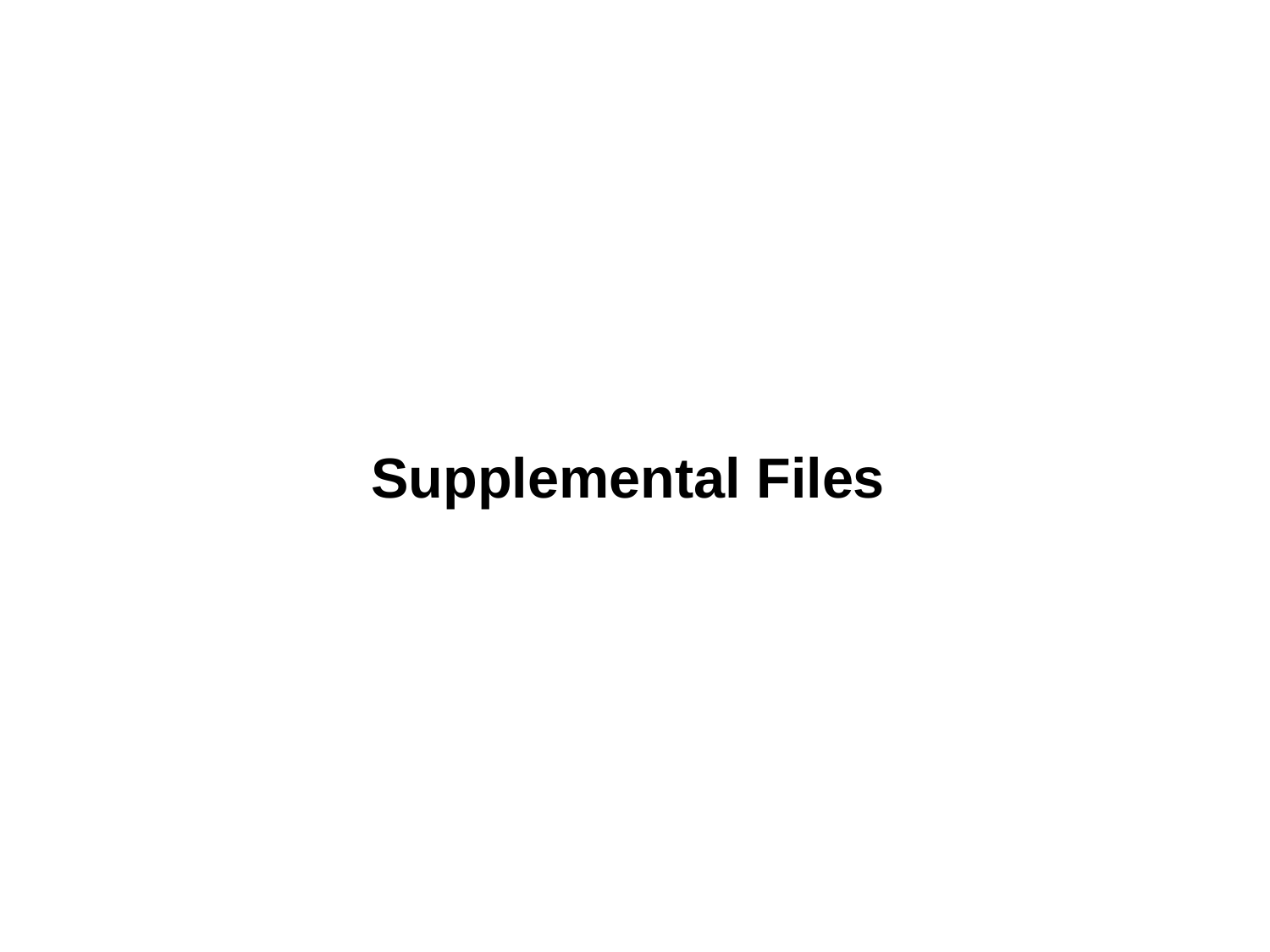

Supplemental Files

## Slide 2
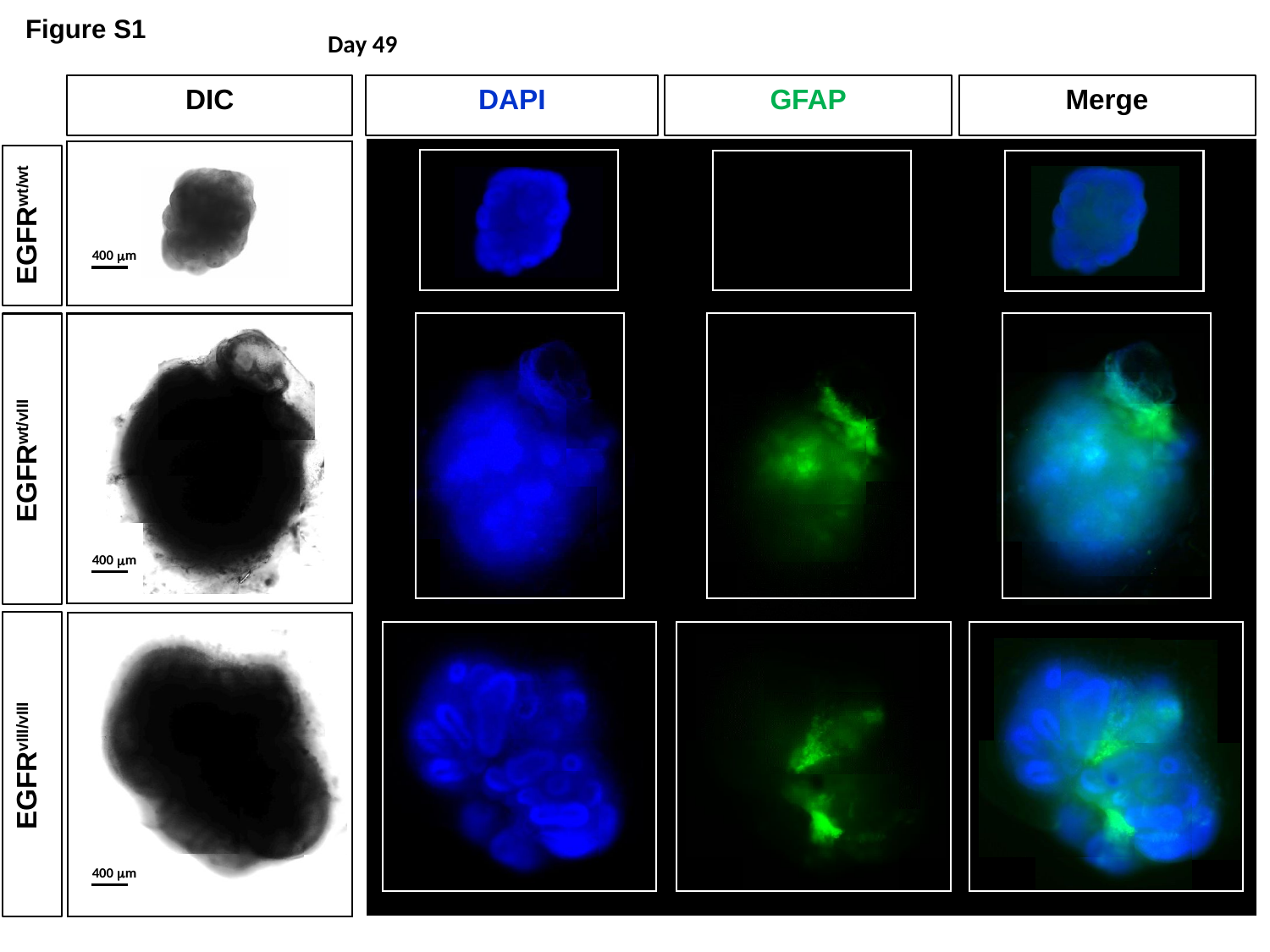

Merge
DAPI
GFAP
DIC
Figure S1
Day 49
EGFRwt/wt
400 mm
EGFRwt/vIII
400 mm
EGFRvIII/vIII
400 mm

## Slide 3
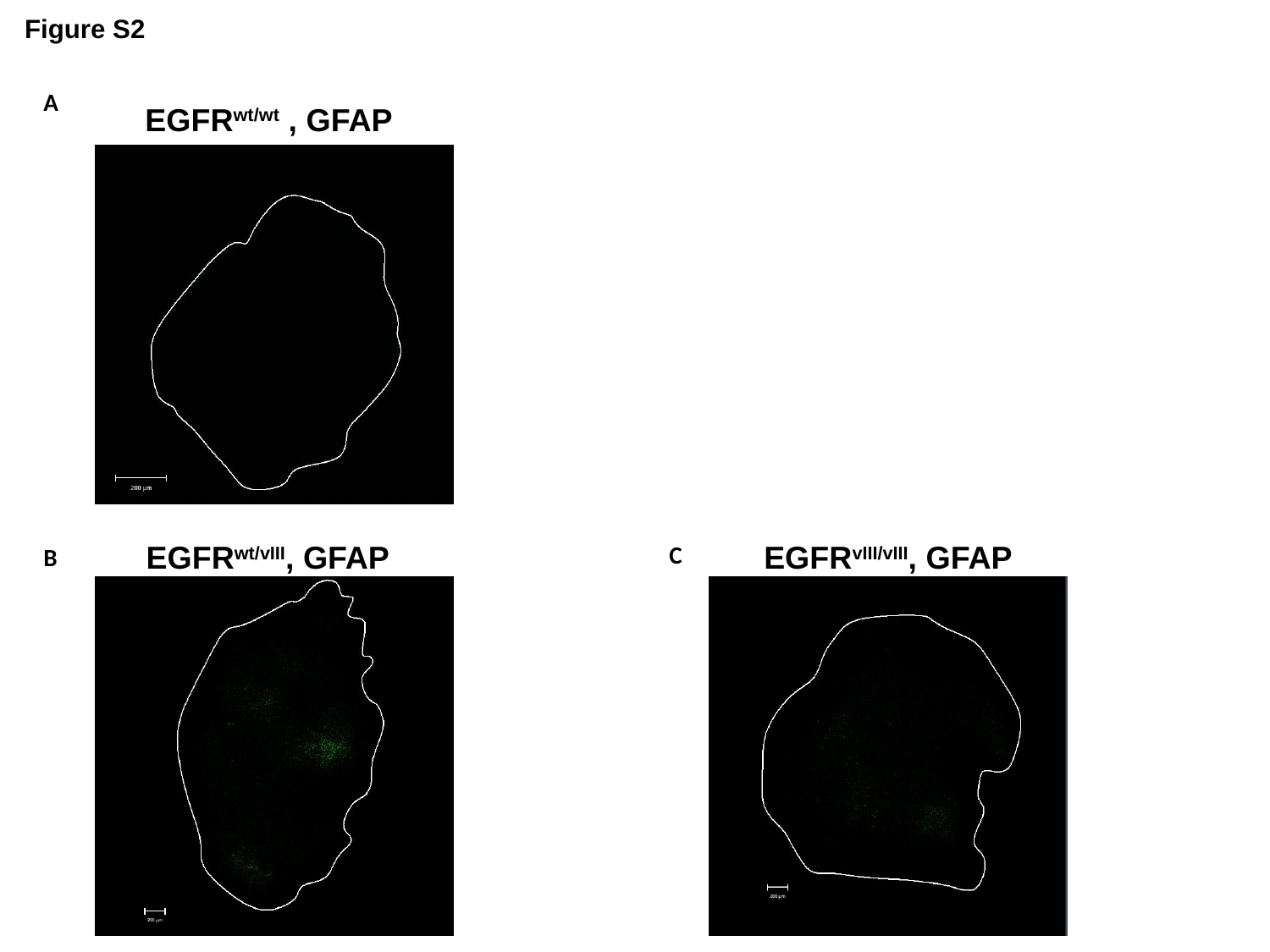

Figure S2
A
EGFRwt/wt , GFAP
EGFRwt/vIII, GFAP
EGFRvIII/vIII, GFAP
C
B

## Slide 4
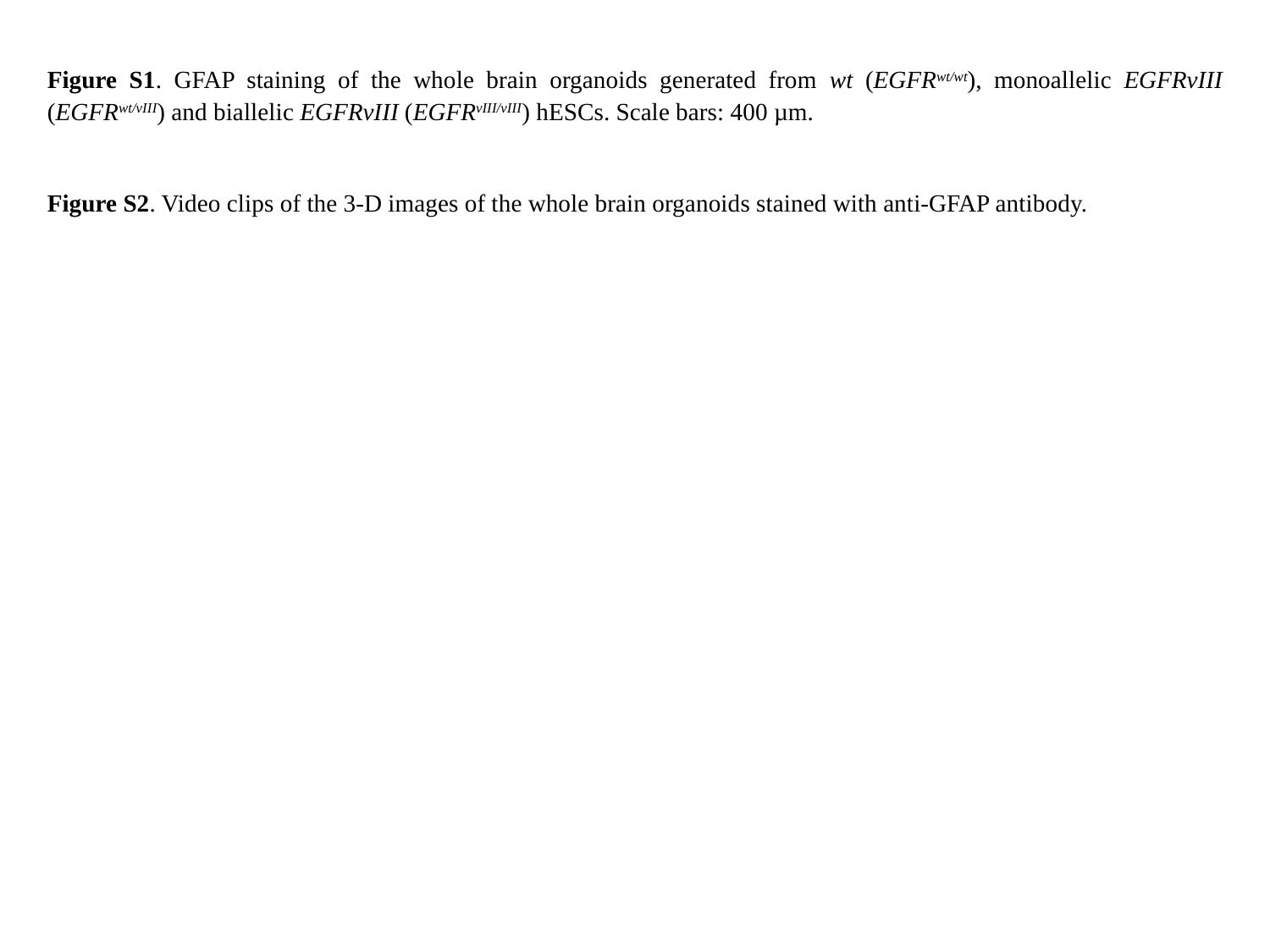

Figure S1. GFAP staining of the whole brain organoids generated from wt (EGFRwt/wt), monoallelic EGFRvIII (EGFRwt/vIII) and biallelic EGFRvIII (EGFRvIII/vIII) hESCs. Scale bars: 400 µm.
Figure S2. Video clips of the 3-D images of the whole brain organoids stained with anti-GFAP antibody.
