# Supplementary material for: The epidermal growth factor receptor variant type III mutation frequently found in gliomas induces astrogenesis in human cerebral organoids
Source: Cell Prolif. 2020 Dec 6;54(2):e12965. doi: 10.1111/cpr.12965 (PMC7848959; doi:10.1111/cpr.12965)
Supplement: Supplementary file 3 — Table S2 [file CPR-54-e12965-s003.docx]

Table S2. List of antibodies used in this study

| Antibody | SOURCE | IDENTIFIER |
| --- | --- | --- |
| Mouse monoclonal anti-SOX-2 | Millipore | Cat# MAB4343, RRID: AB_827493 |
| Rabbit polyclonal anti-PAX-6 | BioLegend | Cat# 901301, RRID: AB_2565003 |
| Mouse monoclonal anti-NeuN | Millipore | Cat# MAB377, RRID: AB_2298772 |
| Rabbit polyclonal anti-MAP2 | Millipore | Cat# AB5622, RRID: AB_91939 |
| Mouse monoclonal anti-Nestin | Millipore | Cat# MAB5326, RRID: AB_11211837 |
| Mouse monoclonal anti-TUJ1 | Convance | Cat# MMS-435P, RRID: AB_2313773 |
| Mouse monoclonal anti-GFAP | Thermo Fisher Scientific | Cat# MA5-12023, RRID: AB_10984338 |
| Rabbit monoclonal anti-GFAP | Abcam | Cat# ab68428, RRID: AB_1209224 |
| Rabbit polyclonal anti-S100β | Novus Biologicals | Cat# NBP1-87102, RRID: AB_11044221 |
| Mouse monoclonal anti-OCT4 | Santa Cruz | Cat# sc-5279, RRID: AB_628051 |
| Mouse monoclonal anti-SSEA4 | Santa Cruz | Cat# sc-21704, RRID: AB_628289 |
| Mouse monoclonal anti-TRA1-81 | Millipore | Cat# MAB4381, RRID: AB_177638 |
| Mouse monoclonal anti-SMA | Agilent | Cat# M0851, RRID: AB_2223500 |
| Rabbit polyclonal anti-AFP | Agilent | Cat# A0008, RRID: AB_2650473 |
| Mouse monoclonal anti-Ki67 | BD Biosciences | Cat# 550609, RRID: AB_393778 |
| Rabbit polyclonal anti- Cleaved Caspase-3 | Cell Signaling | Cat# 9661, RRID: AB_2341188 |
| Goat anti-mouse Alexa Fluor 488 | Thermo Fisher Scientific | Cat# A11001; RRID: [AB_2534069](http://antibodyregistry.org/AB_2534069) |
| Goat anti-rabbit Alexa Fluor 594 | Thermo Fisher Scientific | Cat# A11012; RRID: [AB_2534079](http://antibodyregistry.org/AB_2534079) |
| Goat anti-rabbit Alexa Fluor 488 | Thermo Fisher Scientific | Cat# A-11008, RRID: AB_143165 |
| Goat anti-mouse Alexa Fluor 594 | Thermo Fisher Scientific | Cat# A-11005, RRID: AB_141372 |
